# Core Verbal Autopsy Procedures with Comparative Validation Results from Two Countries

**DOI:** 10.1371/journal.pmed.0030268

**Published:** 2006-07-18

**Authors:** Philip W Setel, Chalapati Rao, Yusuf Hemed, David R Whiting, Gonghuan Yang, Daniel Chandramohan, K. G. M. M Alberti, Alan D Lopez

**Affiliations:** 1 MEASURE Evaluation/Carolina Population Center, University of North Carolina, Chapel Hill, North Carolina, United States of America; 2 Department of Epidemiology, University of North Carolina, Chapel Hill, North Carolina, United States of America; 3 School of Population Health, University of Queensland, Herston, Australia; 4 Tanzania Ministry of Health, Dar es Salaam, Tanzania; 5 University of Newcastle upon Tyne School of Clinical Medical Sciences, Newcastle upon Tyne, United Kingdom; 6 Center of Non-Communicable Diseases and Behavioral Risk Factor Studies, Chinese Academy of Medical Sciences, Beijing, China; 7 Department of Infectious and Tropical Diseases, London School of Hygiene and Tropical Medicine, London, United Kingdom; 8 Department of Endocrinology and Metabolism, Imperial College London, London, United Kingdom; University of the Witwatersrand, South Africa

## Abstract

**Background:**

Cause-specific mortality statistics remain scarce for the majority of low-income countries, where the highest disease burdens are experienced. Neither facility-based information systems nor vital registration provide adequate or representative data. The expansion of sample vital registration with verbal autopsy procedures represents the most promising interim solution for this problem. The development and validation of core verbal autopsy forms and suitable coding and tabulation procedures are an essential first step to extending the benefits of this method.

**Methods and Findings:**

Core forms for peri- and neonatal, child, and adult deaths were developed and revised over 12 y through a project of the Tanzanian Ministry of Health and were applied to over 50,000 deaths. The contents of the core forms draw upon and are generally comparable with previously proposed verbal autopsy procedures. The core forms and coding procedures based on the International Statistical Classification of Diseases (ICD) were further adapted for use in China. These forms, the ICD tabulation list, the summary validation protocol, and the summary validation results from Tanzania and China are presented here.

**Conclusions:**

The procedures are capable of providing reasonable mortality estimates as adjudged against stated performance criteria for several common causes of death in two countries with radically different cause structures of mortality. However, the specific causes for which the procedures perform well varied between the two settings because of differences in the underlying prevalence of the main causes of death. These differences serve to emphasize the need to undertake validation studies of verbal autopsy procedures when they are applied in new epidemiological settings.

## Abstract 2

A procedure for recording verbal autopsy information was tested in two countries and found to be capable of providing reasonable mortality data. The need to undertake validation studies was also demonstrated.

## Editors' Summary

**Background.:**

People living in developed countries take it for granted that when a loved one dies an accurate cause-of-death certificate will be issued. But for two-thirds of the deaths that occur worldwide, there are no certificates. Detailed information about what people die from is unavailable for more than 50% of countries, many of which have high death rates. This information is badly needed for public-health planning, for using scarce health resources wisely, and for monitoring the effect of new health initiatives. One way to improve knowledge about what people die from is a procedure called verbal autopsy (VA). Relatives or caregivers are interviewed about the symptoms experienced by the deceased before their death and the circumstances surrounding their death by trained personnel who use a standard form. Doctors then review the completed VA forms and assign a specific cause of death from a short version of the International Classifications of Diseases, or ICD, an internationally agreed on list of codes for hundreds of diseases.

**Why Was This Study Done?:**

VA procedures are being developed in many countries, but each step in a VA can be affected by factors that vary from place to place, such as how long after the death the interview is done, the training that interviewers receive, how the questions are worded, and the locally common diseases, which tend to be recognized better than rare diseases. To ensure that the data collected are accurate and comparable between countries and also over time, VA procedures need to be standardized. In this study, the researchers describe their efforts to achieve this through the development and validation of core VA procedures.

**What Did the Researchers Do and Find?:**

In 2001, the researchers refined the VA forms that were being used in Tanzania for deaths occurring around the time of birth and for deaths occurring in childhood and adulthood. They then translated the forms for use in China, adapting them slightly to allow for cultural differences in how symptoms are described. They also drew up a short list of ICD codes to use in tabulating and validating important causes of death. Then, for four years, they collected VA and medical record information for the same deceased individuals and measured how well the VA procedure agreed with the medical record information in both countries. They found that the procedure could be transferred between China and Tanzania but that it performed rather differently for different causes of death in the two countries. So, in both countries, the procedure accurately recorded tuberculosis, cerebrovascular diseases such as strokes, and transport accidents as causes of death. But some other causes of death were accurately recorded in one country only—generally the common diseases in that country—and many causes of death were inaccurately reported in both countries.

**What Do These Findings Mean?:**

The researchers use their experience of developing VAs for use in Tanzania and China and the results of this study to make several recommendations about how to develop standardized VA procedures that will yield accurate cause of death. For example, they suggest that the VA form should contain a detailed core symptom duration checklist and only a short space for a narrative history (an open-ended description of the last illness provided by the relative or caregiver) because long narrative histories are hard to standardize. They discuss the need to adapt core VA forms when moving between countries to allow for linguistic differences and colloquial expression and also the need to consider cultural differences between countries—for example, how soon after bereavement a VA interview can occur. Most importantly, they strongly recommend that validation studies like theirs should be routinely done when VA procedures are applied in new countries or if the major cause of death in a country changes because of a new epidemic or health initiative. Provided this is done, write the researchers, although VA procedures can never be as accurate as proper medical certification at the time of death, they should provide important information about the causes of death for the many countries where this information would otherwise be completely missing.

**Additional Information.:**

Please access these Web sites via the online version of this summary at http://dx.doi.org/10.1371/journal.pmed.0030268.

• World Health Organization information on mortality and on the International Classification of Diseases

• The United Nations' World Mortality Report 2005

• Information on the Tanzania Ministry of Health Adult Morbidity and Mortality Project, which used the VA procedures on which this study was based

• A description of a standard VA method for investigating deaths in infants and children from the World Health Organization

• The INDEPTH Network, an organization collecting health statistics from developing countries that provides standardized VA forms

• MEASURE Evaluation, a USAID-funded project that, in collaboration with the US Census Bureau and the University of Queensland (Australia), supports countries to implement core VA procedures and sample/sentinel vital registration methods

• The Health Metrics Network, a global collaboration focused on strengthening country health information systems to generate sound data for decision-making at country and global levels, is committed to improving sources of vital statistics and cause-of-death data

## Introduction

Globally, only about a third of all deaths are registered with age, sex, and cause [1]. The vast majority of these are in developed countries. In sub-Saharan Africa, where premature mortality accounts for about 80% of the total burden of disease [2], the need to remedy this situation is urgent. What is known about causes of death in these areas comes primarily from demographic surveillance sites and is largely limited to causes of death among children [3–6]. While more is known about child mortality than that of adults, knowledge remains patchy for neonatal and perinatal mortality [7].

In a 2003 address to the World Health Organization (WHO) staff, Director General Jong-Wook Lee succinctly highlighted the urgency of improving knowledge about vital events: “To make people count, we first need to be able to count people” [8]. The WHO has likened the continued lack of quality health information in lower-income countries, including data on vital events, to a “gathering storm” [9]. The crisis is being precipitated by the rapid escalation in national data demands and in reporting requirements for international initiatives, many of which require summary measures of survival and/or cause-specific mortality as indicators of program impact.

Improving the monitoring of vital events, and generating representative mortality statistics in lower-income countries in particular, will require new techniques, new technologies, and new thinking about sustainable, representative, and reliable systems for registering deaths and determining causes [10].

Sample or sentinel mortality surveillance using standardized “verbal autopsy” (VA) procedures represents a viable mid- or long-term strategy for improving mortality information [10]. A VA is an interview administered to caregivers or family members after a death occurs. A wide range of interview instruments and cause-of-death attribution procedures have been developed for this purpose [11–18]. Although VA is a limited tool [19], the procedure has demonstrated the ability to produce valid estimates of the mortality cause structure in many settings [14,20–25]. Some assessments of the validity and cross-comparability of VA-derived mortality estimates for child mortality have also been conducted [4–6]. VA has been applied in numerous countries, among children and adults, and for the purposes of both exploring specific causes of death in research projects and developing an overall description of the mortality structure at the community or population level. The WHO and the United Nations Children's Fund have called for the expanded use of the technique to monitor child mortality for at least a decade [26].

This article presents a proposed set of core VA procedures and the summary results of a two-country validation study conducted in Tanzania and China. These procedures are proposed for adaptation to a variety of settings, particularly in the context of sample or sentinel vital registration.

Experiences from India [27], China [28], and Tanzania [29,30] have shown how information generated through community-based mortality surveillance using VA can influence health policy, practice, monitoring, and evaluation. Generating data from VA procedures follows a simple, stepwise process. First, deaths are registered using some form of active, community-based reporting system. Second, VA interviews are obtained by trained interviewers who visit the households of the deceased within a specified period after the death. Third, physician certifiers use these completed VA interview forms to assign a specific cause of death, and write death certificates according to protocols based on the *International Statistical Classification of Diseases and Related Health Problems,* 10th revision (ICD-10) [31–33]. Lastly, mortality data are tabulated on a periodic basis and fed into routine reporting, planning, and monitoring processes and are used to analyze mortality structures, levels, and trends.

The ultimate impact of mortality surveillance will hinge upon the validity, comparability, and consistency of tools and methods used to obtain the “raw” data from representative sample or sentinel populations. In order to contribute to the expanded use of VA in sample and sentinel registration, as well as in research, this paper proposes a core set of VA procedures that have been validated in China and Tanzania. Where relevant, we have compared these procedures with those used in other settings.

## Methods

Between 1992 and 2004, the VA procedures presented here were developed as part of a long-term national system of sentinel demographic surveillance in Tanzania. The forms, coding methods, and mortality surveillance activities were integrated into the routine functions of local health authorities in Tanzania [34–36] and were applied in more than 50,000 deaths. In 2001, the procedures were further refined with reference to other existing and recommended tools. They were then translated with additional slight modifications for use in the Chinese Disease Surveillance Points System and vital registration system. The Chinese Disease Surveillance Points System is China's national sample vital registration system, covering 6% of the population in 160 urban and rural clusters [37].

The procedures discussed in this article were the subject of a 4-y validation study in Tanzania and China. The details of the study protocol and results for all age groups from both countries have been published elsewhere [38,39]. Briefly, the protocol entailed collection of VA and medical record information for the same individuals. In Tanzania, data were collected from urban and rural sentinel demographic surveillance areas operated by the Tanzanian Ministry of Health through the Adult Morbidity and Mortality Project, and from nearby health facilities [40]. Deaths from the sentinel areas were eligible for inclusion if the deceased visited a health facility during the period during which the “terminal” events leading to the death occurred. This did not necessarily mean that the death took place in the facility. For all eligible deaths an attempt was made to trace the medical records after informed consent was obtained from surviving family members. For deaths that occurred in participating health facilities during the study period, all were eligible, provided they met a geographic restriction criterion to ensure comparability with deaths from the sentinel surveillance sites [38]. In these cases, the medical records were obtained from the health facility, and the relatives, if they gave consent, were traced to their homes, usually within 1 mo after the death, and a VA interview was administered. In China, data were collected from urban areas through collaboration with the national Disease Surveillance Points System. Deaths were included from 100 tertiary hospitals in six cities. The number of deaths selected for each cause was based on the frequency in the routine system, with oversampling of some rarer causes and undersampling of some very common causes.

Physician panels assigned causes of death to all VAs and medical records using standard procedures, including blinding. Medical records and VA data were handled identically in this regard, and both sources were used to produce standard death certificates. No physician assigned causes of death using both the VA and medical record for the same individual. Entries were then coded to ICD-10 at the core code and four-digit levels and tabulated according to the list in Table 1. The table contains a 57-item VA tabulation list with ICD-10 core codes in the third column. The list is organized according to International Statistical Classification of Diseases (ICD) principles, and contains the causes that are amenable to detection by VA and are relevant for guiding policy and program development. It is important to use such a tabulation list as a minimum standard for reporting in order to maintain international comparability of mortality datasets.

**Table 1:**
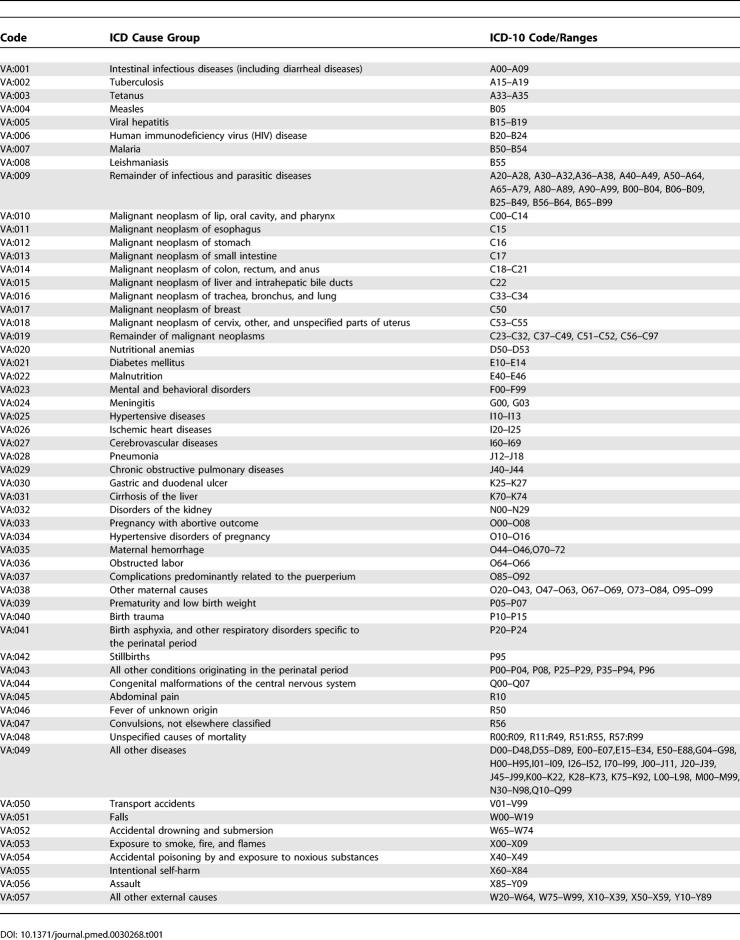
ICD Mortality Tabulation List for VA Data

## Results

### Content of Core VA Interview Forms

In order to function well as part of routine systems, the forms had to be easy to use by interviewers with varying degrees of clinical skills and knowledge. They were also used to record relevant contextual information (such as use of health facilities in the period before death and data on risk factors). Additionally, the forms made use of any documentary evidence available from the household of the deceased that might aid in determining the probable cause of death. Lastly, the forms had to provide physicians with enough data to produce internationally comparable mortality statistics based on ICD coding guidelines, and be amenable to developing data-derived algorithms to determine the probable cause of death [41].

All short core VA forms referred to in this article are available online. They include the forms for perinatal events and neonatal deaths (Figure S1), deaths in post-neonatal children under age 5 (Figure S2), and deaths among persons aged 5 y and above (Figure S3). Each form follows the same basic structure: identifying information about the deceased (including age, sex, and place of death), cause of death according to respondent, short narrative history, symptom duration checklist, health services used in the period before death, and any medical evidence available at the household, including whether a health worker informed the respondent of the cause of death.

A section on the condition of the mother during and after pregnancy and birth is included on the neonatal form. Deaths to women of reproductive age, and maternal deaths in particular, are addressed in a subsection of the form for deaths over age 5. The questions contained in the symptom duration checklist are generally arranged by anatomical system. They are intended to provide strong support for a positive diagnosis of probable cause of death, and the confident exclusion of differential diagnoses.

Table 2 compares the VA form used at some sites that are members of the INDEPTH Network [42], the VA form used by the Indian Sample Registration System (Indian SRS) [15–18], and a VA form from the WHO [12], and notes key areas of difference in content. VA interview forms tailored to specific age groups generally use the same standard cut-off for neonatal mortality (i.e., death before 28 d), although there is some variation in the age range for the application of forms for post-neonatal child deaths [17,42–44]. For reasons of cost and ease of implementation, the layout and length of the proposed core forms were limited to no more than two A4-sized pages. In this they are similar to forms used in the Indian SRS, and much briefer than most other VA forms presented in the literature [12,13,41].

**Table 2:**
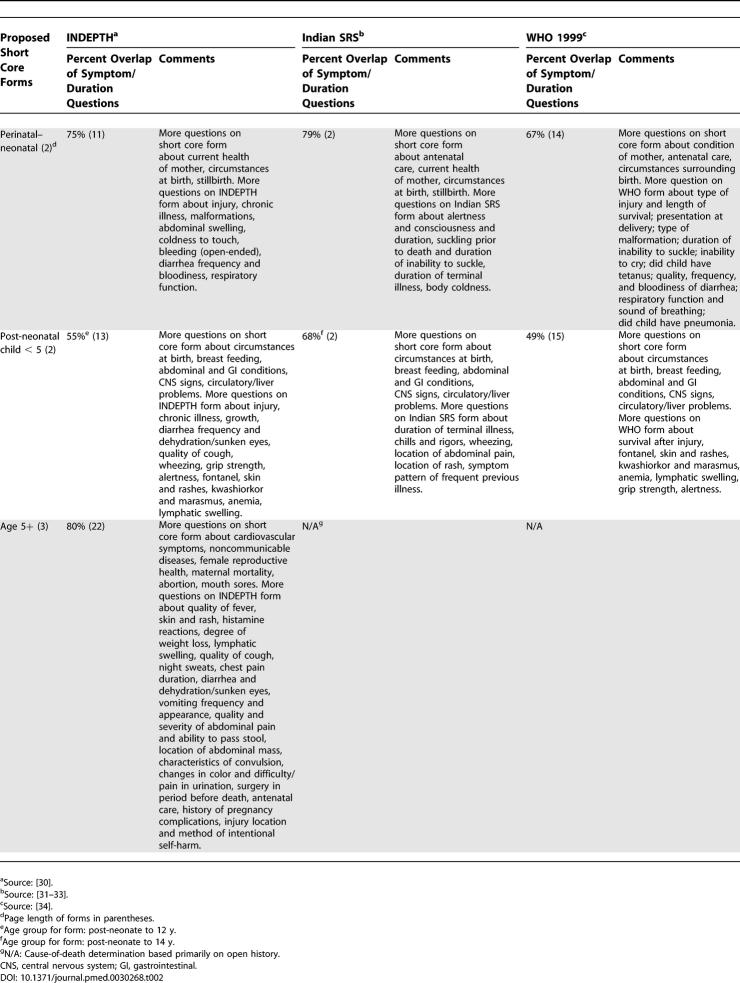
Content Comparison of Verbal Autopsy Forms

Aside from the differences noted in Table 2, the use of long “open history” sections in other forms is another major difference between the proposed core forms and other published VA tools. The Indian SRS form for adult deaths, for example, relies almost exclusively on narrative histories of the events preceding death to provide evidence about the cause of death. If administered as a clinical history, these sections can provide relevant information to physicians who assign probable causes of death. The short core forms in Figures S1–S3 allow for brief narrative histories, but emphasize a “symptom duration checklist” approach for use in cause-of-death attribution.

Experience in implementing VA procedures suggests that lengthy clinical history sections cannot be standardized and vary substantially depending on the clinical skills and medical training of the interviewer. In addition, interviewers may introduce bias into data collection by recording histories that neatly fit into known or familiar disease descriptions or are based on the interviewers' initial impressions of the likely cause of death. Therefore, a core symptom duration checklist may be more systematic and to produce a more complete inventory of the signs and symptoms before death than would a heavier reliance on open histories.

### Interviewing Protocols and Cross-Cultural Applicability of Procedures

A VA interview is conducted similarly to any confidential health-related interview, with the added consideration that the subject matter concerns a topic that could hardly be more distressing—the recent death of a family or household member. This, in part, speaks to the need to enroll respected community members in areas where VA will be implemented to help build local awareness and acceptance of what is, generally speaking, a new and unfamiliar mechanism of collecting health information.

There is a range of opinion about whether medical training should be a preferred qualification for VA interviewers or whether educated but non-medically trained persons are more suitable. Local experience will determine the optimal solution. Training should include discussion of symptoms and their description in local languages. In addition, a clear understanding of how live births and stillbirths can be accurately differentiated using appropriate terminology is important. Ideally, the interview should happen as soon as possible after a death with due consideration to culturally appropriate mourning periods. All questions on the VA form (aside from the appropriate skips) must be asked of the respondent regardless of the opinion of the interviewer as to their relevance. Quality assurance should be performed routinely. If feasible, re-interview of a 10%–15% sample of VAs would offer a strict standard. However, given the sensitive nature of VA, it may be sufficient to verify for a similar proportion of deaths that (a) the death indeed occurred and (b) the VA interview in fact took place at the household of the deceased, with an appropriate respondent. Interviewer retraining and supportive supervision are probably the most important components of quality assurance for VA.

As with any survey instrument intended for cross-cultural and cross-linguistic application, care must be taken in translation into local languages and field testing so that all questions are understood by respondents in the way they are intended [22]. Part of the cultural validation of VA should include observations of VA by a medical anthropologist or sociologist, and interviews with community members to ensure accurate understanding of terms used in the VA form. VA interviewers should be informed about any areas of potential confusion due to local or colloquial expressions. This will help ensure that in addition to building community rapport for administering VA, culturally appropriate and sensitive terms, idioms, and expressions are used in interviews without sacrificing precision and cross-comparability of results. A balance must be struck between clinically precise terminology, which can be confusing or even offensive in the context of a VA interview, and colloquial expressions or local terms that might impede accurate cause of death attribution and ICD coding.

It is also important to consider linguistic and cultural issues in implementing proposed core VA forms in very different settings. The experience of transferring these procedures from Tanzania to China has been instructive in this regard. It is felt that both the brevity of the interviews and the efforts expended in establishing rapport contributed to attaining response rates in both settings of over 90%. Minimal, though important, modifications were required to translate specific questions and variables from the original Tanzanian forms into the Chinese context. For instance, the question “Was [the deceased] breathless on lying flat?” employed in Tanzania was not clearly interpreted in China, and a question on “breathlessness interfering with sleep” was substituted. Chinese interviewers readily adopted the protocols, and physician reviewers in both sites were able to certify causes of death using an international death certificate. Finally, statistics could be compiled from both countries according to the proposed tabulation list, yielding internationally comparable data.

### ICD Coding, Cause-of-Death Attribution, and Tabulation List

Because VA may serve as the best or even sole evidence on cause of death in many settings, establishing international comparability is important [4–6,26,45]. The lack of standard interview forms, cause-of-death categories, and coding practices has hindered attempts to synthesize results from various applications of VA to assessing precise causes of child mortality [4,6,45]. Therefore, it is recommended that the causes of death as determined through physician review of VA be recorded using a four-line death certificate (i.e., underlying, immediate, associated, and contributory causes). Subsequently, a physician or medical recorder should select and code the underlying cause to the core 3 character code using standard ICD-10 rules.

In certain cases it may be possible to code to the fourth digit. On the other hand, ICD-10 rules may frequently preclude the use of certain three-digit codes in the VA context. For example, ICD codes starting with B50, B51, and B52 refer to malaria. Use of these codes requires both confirmation of parasite infection and identification of the malaria species. It is unlikely that evidence of such confirmation would be available at the household level. In such instances there are usually three-digit codes available for use (in this case either “B53 other parasitologically confirmed malaria” or “B54 unspecified malaria—clinically diagnosed malaria without parasitological confirmation”) that would not affect the outcome of tabulating and reporting VA data, or the main public-health interpretations and policy implications of the tabulated data.

Physicians who review the completed VA forms usually require training in cause-of-death certification using ICD rules and international death certificates. An explanation of the structure and content of the ICD classification, and of the rules for selection and coding of the underlying cause of death, is also necessary to ensure uniformity of data across different coders. Details of coding guidelines and criteria, manuals, and options for organizing VA coding can be obtained from the authors.

The issue of reporting single versus multiple causes of death in VAs, particularly for children, has been addressed extensively. Most sources recommend or employ multiple cause-of-death attribution in children without providing a single underlying cause [43,44,46–48], and at least one source does so for adults [13]. We recommend the use of standard death certificates for all ages in accordance with ICD convention ([32], p. 31). This enables recording, coding, and analysis of multiple causes of death while retaining comparability of mortality data based on the tabulation of a single underlying cause, as prescribed by ICD. Although ICD does recommend a specially designed death certificate for perinatal deaths ([32], p. 90), few countries have implemented it. For the present, therefore, perinatal deaths (and stillbirths, if desired) may be recorded, together with neonatal deaths, on a conventional death certificate.

The ICD recommends two “condensed” tabulation lists for mortality reporting [33]. These lists contain many causes that can be accurately identified only with specific diagnostic or clinical information. In the case of VA, the smaller list of causes presented in Table 1 is more appropriate. The ICD specifically sanctions the development of such tailored tabulation lists [32].

### Validation and Comparative Findings from China and Tanzania in Deaths over Age 5

There were 25 causes in China and 26 causes in Tanzania (for at least one age group) for which there were at least five deaths in both the VA and the medical records. Table 3 shows summary results for causes of death for which at least five deaths were validated, sensitivity was greater than 50%, and the relative difference in the cause-specific mortality fraction (CSMF) in the VA (CSMF_VA_) and medical record (CMFS_MR_) was equal to or less than 20%. These criteria are based on threshold values for sensitivity and CSMF suggested for assessing accuracy of adult VA [49]. The comparison of CSMFs was based on the relative difference in the proportion of deaths due to cause *X* in the medical records dataset (CSMF_MR_) from the proportion of deaths due to the same cause in the verbal autopsy dataset (CSMF_VA_). We calculated sensitivities and specificities for all causes using conventional two-by-two table analysis, although results are displayed only for those causes reaching the threshold sensitivity. For the over-five age group, data were available for 1,912 deaths in 42 cause-of-death categories from Tanzania, and 2,029 deaths in 37 categories from rural areas in China. Of these, 140 deaths from Tanzania and 170 deaths from China were coded to either “all other specified diseases” or “undetermined”; these are excluded from the comparison.

**Table 3:**
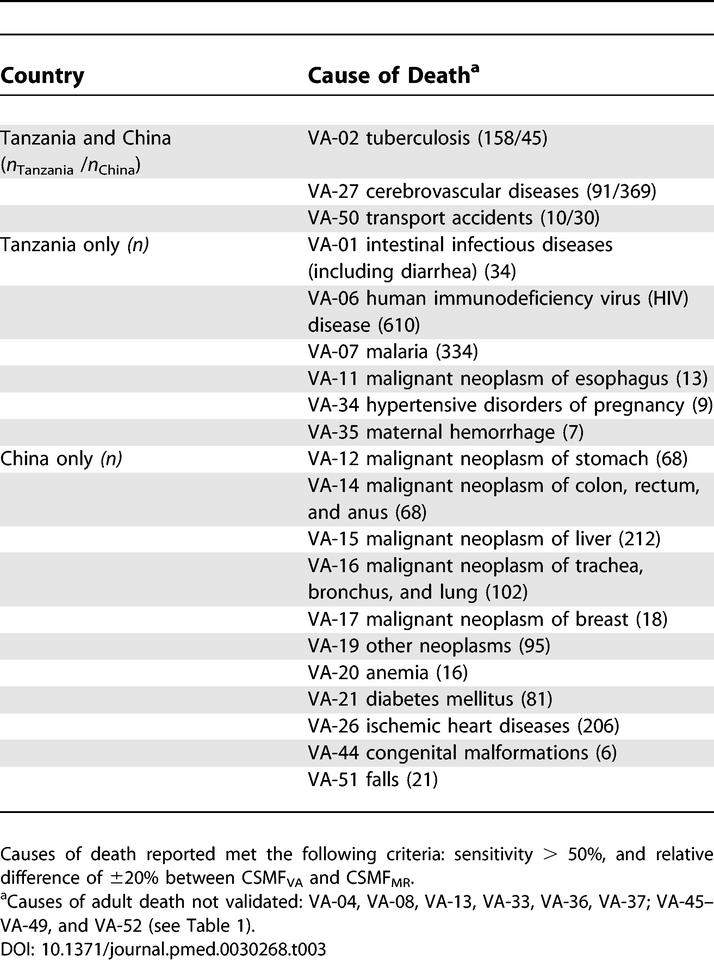
Validation Results for Causes of Adult Mortality in Tanzania and China

Of the 20 causes of death listed in Table 3, three (VA-02 tuberculosis, VA-27 cerebrovascular diseases, and VA-50 transport accidents) met the threshold criteria in both countries. Six causes reached the threshold in Tanzania only, and 11 causes met the threshold levels in China only. At the upper bound of the 95% confidence level for sensitivity, seven additional causes reach the threshold in Tanzania (VA-09 remainder of infectious and parasitic diseases, VA-15 malignant neoplasm of liver, VA-19 other neoplasms, VA-21 diabetes mellitus, VA-25 hypertensive diseases, VA-38 other maternal causes, and VA-57 all other external causes).

Significantly, for six causes (VA-09 remainder of infectious and parasitic diseases, VA-17 malignant neoplasm of breast, VA-21 diabetes mellitus, VA-25 hypertensive diseases, VA-29 asthma/chronic obstructive pulmonary disease, and VA-51 falls) the relative difference in sensitivity of VA was less than 25%. It should be born in mind that because of the small samples for certain causes of death it was not possible in the studies to validate all the causes contained in Table 1.

## Discussion

The increasing importance of VA is reflected in the growing number of meta-analyses of VA-based datasets on child mortality from demographic surveillance sites and special studies [4–6,45], all of which make the case for standardized procedures. The procedures presented here are the product of over a decade of application, trial, assessment, and refinement, and have considerable commonalities with other forms in the public domain.

The main purpose of these tools is to supply countries that have no source of reliable mortality reporting and cause-of-death data with the means to confidently produce and use accurate, repeatable, and internationally comparable measurements of the cause structure of mortality for the most important diseases and conditions, and that are free from major systematic misclassification. To be sure, VA is a crude substitute for proper medical certification of cause of death—which can be a dubious “gold standard” even in developed countries [50]. The Tanzania–China experience has shown that the transfer of this technology from one setting to another is feasible and can produce results with acceptable sensitivity and CSMFs for important causes of death. In wider application, local validation studies should be considered an essential part of implementing VA procedures intended for national monitoring, evaluation, priority-setting, and policy-making.

These VA procedures performed quite differently for different causes in China and Tanzania. In Tanzania, where more data were available to analyze VA performance in younger age groups (analysis not presented), VA yielded good sensitivity, specificity, and CSMFs for several important causes including pneumonia, but did not perform as well for others, including childhood malaria. This re-emphasizes the need to bear in mind previous findings that both the number of different causes and their underlying prevalence vary by age and across settings where the use VA procedures is appropriate, and that this variation affects VA performance [19,51,52]. Thus, wherever feasible, VA procedures should be accompanied by a validation study, and revalidation should be undertaken periodically if there are indications of major shifts in causes of mortality—either as a result of successful large-scale intervention, or due to epidemics. Validation studies should also take into consideration that the “gold standard” of medical record diagnosis is often an imperfect one, at best. A carefully conducted VA may be superior to poorly maintained or scanty medical records, as seen, for example, in stillbirth [53]. It should also be acknowledged that for some conditions, such as malaria mortality among adults, for which no reliable statistics exist, neither VA nor medical records may form a suitable evidence base.

In addition to the use of standard procedures, the following are needed in order to make the best informed use of VA: further validation studies for less prevalent causes and whenever the procedures are applied in a new setting, further investigation into the effect of recall period [54] and respondent characteristics (e.g., relationship to the deceased and education), further development of guidelines and criteria for assigning cause of death, systematic handling of misclassification error, and candor with respect to any insuperable limitations of VA [14,19]. The WHO's leadership in the future development of VA procedures will be critical to establishing international standards.

Even with extensive validation, the weight given to VA-derived mortality data is likely to be an ongoing topic of debate. Ultimately, the interpretation of how well VA performs is entirely dependent upon the function the technique is meant to perform. VA will never meet the standards of proper medical certification of death at the time of its occurrence. The technique has inherent shortcomings including the prevalence dependency of its accuracy, and the serious effects that variations in sensitivity and specificity can have on comparative estimates of cause-specific mortality across populations or over time in the same population [19,51]. Nevertheless, for purposes of broad priority-setting, tracking trends in mortality due to major conditions of public-health importance, and providing broad burden-of-disease measures, it may be deemed preferable to the current state of near ignorance with regard to direct measures of cause-specific mortality, particularly among adults.

Standard and validated VA procedures are only part of the solution to maximal utility of VA. It is critical to do validation studies so that the degree of uncertainty, which will vary by cause of death, can be factored into mortality burden estimations. While the use of proportional mortality models based on VA data to estimate mortality burdens [4,6,45] remains controversial, these models have the virtue of attempting to make use of the only body of data available on cause-specific mortality for the populations concerned. More significant progress in producing mortality statistics that are valid, comparable, and representative, however, will depend on an expanded commitment to sample vital registration systems that use VA—not through reliance on disease-specific research studies, household surveys, or research demographic surveillance systems. Future research will allow a better understanding of the degree to which these core tools, with locally appropriate modifications, can achieve the ultimate aim of generating reliable and internationally comparable cause-specific mortality statistics.

## Supporting Information

Alternative Language Abstract S1 Translation of the Abstract into French (28 KB DOC)

Alternative Language Abstract S2 Translation of the Abstract into Spanish (29 KB DOC)

Figure S1 Core VA Form 1: Death of Child under 29 d (130 KB DOC)

Figure S2 Core VA Form 2: Death of Child Aged 29 d to under 5 y (129 KB DOC)

Figure S3 Core VA Form 3: Death of Person Aged 5 y and Above (181 KB DOC)

## Abbreviations

CSMF: cause-specific mortality fraction
Indian SRS: Indian Sample Registration System
ICD: *International Statistical Classification of Diseases*
ICD-10: International Statistical Classification of Diseases and Related Health Problems, 10th revision
VA: verbal autopsy
WHO: World Health Organization
