# Supplementary material for: Core Verbal Autopsy Procedures with Comparative Validation Results from Two Countries
Source: PLoS Med. 2006 Jul 18;3(8):e268. doi: 10.1371/journal.pmed.0030268 (PMC1502154; doi:10.1371/journal.pmed.0030268)
Supplement: Supplementary file 1 [file pmed.0030268.sd001.doc]

**Procédures de l'autopsie verbale de noyau avec des résultats comparatifs de validation de deux pays**

***Background*** : Les statistiques cause-spécifiques de mortalité demeurent rares pour la majorité de pays de revenu bas, où les fardeaux de la maladie les plus élevés sont expérimentés. Ni les systèmes d'information service-basés ni l'enregistrement essentiel ne fournissent des données proportionnées ou représentatives. L'expansion de l'enregistrement essentiel d'échantillon avec des procédures l'autopsie verbale représente la solution d'intérim la plus prometteuse pour ce problème. Le développement et la validation des formes verbale de noyau et des procédures appropriées de codage et de tabulation sont une première étape essentielle à prolonger les avantages de cette méthode.

**Méthodes/Principaux résultats** : Des formes de noyau pour le peri- et néonatals, l'enfant, et les décès d'adulte ont été développés et mis à jour sur 12 ans par un projet du Ministère de la Santé Tanzanien et appliqués à plus de les 50.000 décès. Les teneurs des formes de noyau utilisent et sont généralement comparables aux procédures verbales précédemment proposées d'autopsie. Les formes de noyau et les procédures de codage basées sur la classification statistique internationale des maladies (ICD) ont été encore adaptées pour l'usage en Chine. Ces formes, la liste de tabulation d'ICD, le protocole récapitulatif de validation, et les résultats récapitulatifs de validation de Tanzanie et de Chine sont présentés ici.

**Conclusions/Signification** : Les procédures sont capables de fournir des évaluations raisonnables de mortalité comme adjugé contre des critères indiqués d'exécution pour plusieurs causes communes de la mort dans deux pays en structures radicalement différentes de cause de la mortalité. Cependant, les causes de détail pour lesquelles les procédures exécutent bien a changé entre les deux arrangements dus aux différences dans la prédominance fondamentale des causes principales de la mort. Ces différences servent à souligner la nécessité d'entreprendre des études de validation des procédures de VA (autopsie verbale) quand elles sont appliquées dans de nouveaux arrangements épidémiologiques.
