# Supplementary material for: Core Verbal Autopsy Procedures with Comparative Validation Results from Two Countries
Source: PLoS Med. 2006 Jul 18;3(8):e268. doi: 10.1371/journal.pmed.0030268 (PMC1502154; doi:10.1371/journal.pmed.0030268)
Supplement: Supplementary file 2 [file pmed.0030268.sd002.doc]

**Antecedentes:** Las estadísticas de mortalidad causa-especifica siguen siendo escasas para la mayoría de países de ingresos bajos, en donde existe el mayor impacto de enfermedades. Ni los sistemas de información de los establecimientos en salud ni los registros vitales proveen la información adecuada o representativa. La ampliación del registro vital de autopsia verbal representa la solución interino más promisorio para este problema. El desarrollo y validación de los formularios de la autopsia verbal y la codificación y tabulación de los procedimientos son los primeros pasos para extender los beneficios de este método.

**Métodos:** Los formularios de foco de las muertes peri y neonatal, niños y adultos fueron desarrollados, revisados y aplicados a 50,000 muertes durante un periodo de 12 años en un proyecto del Ministerio de Salud en Tanzania. Los contenidos de dichos formularios generalmente son comparables con los procedimientos de las autopsia verbales previamente realizadas. Los formularios de foco y los procedimientos de codificación están basados en la Clasificación Internacional de Enfermedades (CID) y fueron adaptados para su uso en China. Estos formularios, la lista de tabulación de la Clasificación Internacional de Enfermedades, el protocolo de validación y el resumen de los resultados de validación de Tanzania y la China se presentan en este artículo.

**Conclusiones/Significación:** Los procedimientos presentados son capaces de proveer estimados razonables de mortalidad al comparar los criterios de desempeño establecidos para varias causas comunes de muerte en dos países con estructuras causales de mortalidad radicalmente diferentes. Sin embargo, las causas específicas por el buen desempeño de los procedimientos varían entre los dos medios, principalmente por las diferencias en la prevalencia implícita de las principales causas de muerte. Estas diferencias demuestra la necesidad de hacer estudios de validación de los procedimientos de autopsia verbal (AV) cuando se aplican en medios epidemiológicos nuevos.
