# Supplementary material for: Core Verbal Autopsy Procedures with Comparative Validation Results from Two Countries
Source: PLoS Med. 2006 Jul 18;3(8):e268. doi: 10.1371/journal.pmed.0030268 (PMC1502154; doi:10.1371/journal.pmed.0030268)
Supplement: Supplementary file 3 [file pmed.0030268.sg001.doc]

| **Field site** | | | |
| --- | --- | --- | --- |
| **Household number** | | | |
| **Date of interview** | Day | Month | Year |
| **Interviewer** | | | |
| **Location identifier 1** | | | |
| **Location identifier 2** | | | |
| **Name of reference person** | | | |
| **Was an appropriate respondent found?**  1- Yes 2-No | | | |
| **Relationship of the respondent to the deceased** | 1-Father 2-Mother 3-Spouse  4-Sibling 5-Child  6-Other relative 7-No relation | | |
| **Did you live with the deceased in the period leading to death?** | | 1-Yes  2-No |  |
| **Name of deceased** | | | |
| **Sex**  1-Male 2-Female | **Date of birth** | | |
|  | Day | Month | Year |
| **Place of death** | 1-Home  2-Health facility____________  3-Other | | |
| **Date of death** | Day | Month | Year |

**Residential status**

**The deceased was…**

1 - Resident in the SAVVY area

2 - Body brought home for burial

3 - Home-coming sick

**What do you think was the cause of death?**

**(Write exactly as the respondent tells you.)**

**History of events leading to death**

| **"I would like to ask you some questions concerning symptoms that the deceased had/showed when s/he was ill. Some of these questions may not appear to be directly related to his/her death. Please bear with me and answer all the questions. They will help us to get a clear picture of all possible symptoms that the deceased had."** | |
| --- | --- |
| **SYMPTOMS CONCERNING THE MOTHER** | |
| 1. How is the child’s mother now? | 1-healthy, fine  2-she is sick  3-died 9-don’t know |
| 2. Was it a difficult birth? | 1-yes  2-no 9-don’t know |
| 3. Did the mother have fits before giving birth? | 1-yes  2-no 9-don’t know |
| 4. Did/does the mother have high blood pressure? | 1-yes  2-no 9-don’t know |
| 5. Did the mother have a febrile illness at the time of delivery? | 1-yes  2-no 9-don’t know |
| 6. Did the child’s mother suffer from any of the conditions? | 1-diabetes  2-heart disease  3-TB  4-epilepsy  5-none 9-don’t know |

| **EVENTS DURING THE BIRTH OF A CHILD** | | |
| --- | --- | --- |
| 7. Did the mother have any antenatal care during her pregnancy? | 1-yes  2-no  9-don’t know | |
| 8. Where did the mother give birth? | 1-home  2-health facility  3-in transit  4-other place  9-don’t know | |
| 9. Who assisted the birth? | 1-none  2-untrained TBA  3-trained TBA  4-health professional  9-don’t know | |
| 10. Had the mother received Tetanus Toxoid vaccination (TT)? | 1-yes  2-no  9-don’t know | |
| 11F. If yes, how many TT injections did she receive? | Number | |
| 12. Was the child a… | 1-singleton  2-twin 9-don’t know | |
| 13. Was it a forceps or vacuum delivery? | 1-yes  2-no 9-don’t know | |
| 14. Was it a caesarian delivery? | 1-yes  2-no 9-don’t know | |
| 15. Was it a prolonged labour? | 1-yes  2-no 9-don’t know | |
| 16. Did waters break 1 day or more before delivery of the baby? | 1-yes  2-no  9-don’t know | |
| 17. Was the child premature? | 1-yes  2-no 9-don’t know | |
| 18F. If yes, how many months or weeks? |  | Months |
|  |  | weeks |
| 19. Did the baby play or move in the womb before labour? | 1-yes  2-no 9-don’t know | |
| 20F. If no, did the baby breathe at all after delivery? | 1-yes  2-no 9-don’t know | |
| 21. Was the baby dead at birth? | 1-yes  2-no 9-don’t know | |
| 22. Did the umbilical cord come before the baby was born? | 1-yes  2-no 9-don’t know | |
| **ASK THESE QUESTIONS IF THE**  **CHILD WAS BORN ALIVE** | | |
| 23. Did the child cry immediately after birth? | 1-yes  2-no 9-don’t know | |
| 24. Was the child able to breastfeed soon after birth? | 1-yes  2-no 9-don’t know | |
| 25F. If no, was the problem with the child or the mother? | 1-child  2-mother  9-don’t know | |
| 26. Was the child weighed after being born? | 1-yes  2-no 9-don’t know | |
| 27F. If yes, how much did the child weigh? | | Kgs |
| 28. Were there any bruises or signs of injury on the child’s body after birth? | 1-yes  2-no  9-don’t know | |
| 29. What was the color of the child’s skin after being born? | 1-normal  2-purple  3-pale 9-don’t know | |
| 30. Did the child’s arms/legs have strength? | 1-yes  2-no 9-don’t know | |
| 31. Did the child have any malformation at birth? | 1-yes  2-no 9-don’t know | |
| 32. Did the eye colour change to yellow (jaundice)? | 1-yes  2-no 9-don’t know | |
| 33F. If yes, how many days after being born? | | Days |
| 34. Did the child have any problem with the umbilical cord? | 1-yes  2-no 9-don’t know | |
| 35. Did the child have a fever? | | Days |
| 36. Did the child have convulsions? | 1-yes  2-no 9-don’t know | |
| 37. During the period of illness did s/he have areas of skin that were red peeling or skin rash with blisters containing pus? | 1-yes  2-no  9-don’t know | |
| 38. Was the child coughing? | | Days |
| 39. Did the child have difficulty breathing? | | Days |
| 40F. If yes, did s/he have fast breathing? | | Days |
| 41F. Did s/he have indrawing of the chest while breathing? | | Days |
| 42. Was the child vomiting? | | Days |
| 43. Did s/he have diarrhea? | | Days |
| 44. Was the child unable to breastfeed when s/he was ill? | | Days |
| 45. Was there a bulge in the child’s fontanel? | | Days |
| 46. Did the child have an… | 1-injury  2-accident  3-neither 1 or 2  9-don’t know | |
| 47F. If the answer to question 46 is 1 or 2, what kind of injury or accident? | | |
| 48. During the illness that led to death, did the child become unconscious? | 1-yes  2-no 9-don’t know | |

| **Health services used by the deceased during his/her illness in the period leading to death** | | |
| --- | --- | --- |
|  | | Did s/he get medicine here? |
| Was given traditional medicine | | 1-yes  2-no 9-don’t know |
|  | ? order |  |
| Was given modern medicine | | 1-yes  2-no 9-don’t know |
|  | ? order |  |
| Medicine from family | | 1-yes  2-no 9-don’t know |
|  | ? order |  |
| Went to traditional healer | | 1-yes  2-no 9-don’t know |
|  | ? order |  |
| Village health worker | | 1-yes  2-no 9-don’t know |
|  | ? order |  |
| Government dispensary | | 1-yes  2-no 9-don’t know |
|  | ? order |  |
| Government health centre | | 1-yes  2-no 9-don’t know |
|  | ? order |  |
| Government hospital | | 1-yes  2-no 9-don’t know |
|  | ? order |  |
| Medicine from any government health facility | | 1-yes  2-no  9-don’t know |
|  | ? order |  |
| Private dispensary | | 1-yes  2-no 9-don’t know |
|  | ? order |  |
| Private health centre | | 1-yes  2-no 9-don’t know |
|  | ? order |  |
| Private hospital | | 1-yes  2-no 9-don’t know |
|  | ? order |  |
| Private pharmacy | | 1-yes  2-no 9-don’t know |
|  | ? order |  |
| Didn’t get any service | | 1-no service |

| **Other Evidence and Summary of Details** | | |
| --- | --- | --- |
| Death Certificate | | |
| Cause of death | | |
| Burial Permit | | |
| Cause of death | | |
| Post mortem results | | |
| Cause of death | | |
| MCH/ANC Card | | |
| Hospital prescription forms | | |
| Treatment cards | | |
| Hospital discharge forms | | |
| Diagnosis | | |
| Other hospital documents | | |
| Laboratory/cytology results | | |
|  | No evidence | |
| Did a health care worker tell you the cause of death? | | 1-yes  2-no 9-don’t know |
| *If yes, what did s/he say?* | | |
