# Supplementary material for: Core Verbal Autopsy Procedures with Comparative Validation Results from Two Countries
Source: PLoS Med. 2006 Jul 18;3(8):e268. doi: 10.1371/journal.pmed.0030268 (PMC1502154; doi:10.1371/journal.pmed.0030268)
Supplement: Supplementary file 4 [file pmed.0030268.sg002.doc]

| **Field site** | | | |
| --- | --- | --- | --- |
| **Household number** | | | |
| **Date of Interview** | Day | Month | Year |
| **Interviewer** | | | |
| **Location identifier 1** | | | |
| **Location identifier 2** | | | |
| **Name of reference person** | | | |
| **Was an appropriate respondent found?**  1- Yes 2-No | | | |
| **Relationship of the respondent to the deceased** | 1-Father 2-Mother 3-Spou  4-Sibling 5-Child  6-Other relative 7-No relation | | |
| **Did you live with the deceased in the period leading to death?** | | 1-Yes  2-No |  |
| **Name of deceased** | | | |
| **Sex**  1-Male 2-Female | **Date of birth** | | |
|  | Day | Month | Year |
| **Place of death** | 1-Home  2-Health facility____________  3-Other | | |
| **Date of death** | Day | Month | Year |

**Residential status**

**The deceased was…**

1 – Resident in the SAVVY area

2 – Body brought home for burial

3 – Home-coming sick

**What do you think was the cause of death?**

**(Write exactly as the respondent tells you.)**

**History of events leading to death**

| **"I would like to ask you some questions concerning symptoms that the deceased had/showed when s/he was ill. Some of these questions may not appear to be directly related to his/her death. Please bear with me and answer all the questions. They will help us to get a clear picture of all possible symptoms that the deceased had."** | | |
| --- | --- | --- |
| **SYMPTOMS** | | |
| 1. Was the child small at birth? | 1-yes  2-no 9-don’t know | |
| 2. Was the child born premature? | 1-yes  2-no 9-don’t know | |
| 3F. If yes, how many months or weeks of pregnancy? | months | weeks |
| 4. Was the child breastfeeding? | 1-yes  2-no 9-don’t know | |
| 5F. If yes, did the child stop just before death? | 1-yes  2-no 9-don’t know | |
| 6. Did she have fever? | months | days |
| 7F. If yes, was the fever… | 1-continuous  2-on and off  9-don’t know | |
| 8F. Did s/he have convulsions? | months | days |
| 9. Did s/he have a cough? | months | days |
| 10F. If yes, was the cough… | 1-dry  2-productive  3-with blood  9-don’t know | |
| 11. Did s/he have breathing difficulties? | months | days |
| 12F. If yes, did s/he have fast breathing? | months | days |
| 13F. Did s/he have indrawing of chest while breathing? | months | days |
| 14. Did s/he vomit? | months | days |
| 15F. If yes, did s/he vomit blood? | months | days |
| 16. Did s/he have a mass in the abdomen? | months | days |
| 17. Did s/he have abdominal distension? | months | days |
| 18F. If yes, did the distension start… | 1-suddenly  2-gradually as days went by  9-don’t know | |
| 19. Did s/he have diarrhoea? | months | days |
| 20F. If yes, did s/he have bloody diarrhoea? | 1-yes  2-no 9-don’t know | |
| 21. Did s/he have abdominal pain? | months | days |
| 22. Did s/he have weight loss? | months | days |
| 23. Did s/he have mouth sores? | months | days |
| 24. Did s/he look pale? (on fingers or feet soles) | months | days |
| 25. Did the child’s lips grow darker in color? | months | days |
| 26. Did s/he have puffiness of the face? | months | days |
| 27. Did s/he have swelling of the whole body? | months | days |

| **SYMPTOMS (contd…)** | | |
| --- | --- | --- |
| 28. Did the eye color change to yellow (jaundice)? | months | days |
| 29. Did s/he have ankle swelling? (show that part of the body) | months | days |
| 30. Did s/he have swelling of joints? | months | days |
| 31. Did s/he have measles? | months | days |
| 32. Did s/he have any other skin disease? | months | days |
| 33. Was s/he unusually sleepy? | months | days |
| 34. Did s/he have neck pain? | months | days |
| 35. Did s/he have a headache? | months | days |
| 36. Did s/he develop stiffness of the whole body (before death)? | months | days |
| 37. Did s/he have loss of consciousness? | months | days |
| 38. Did s/he have fits? | months | days |
| 39. Did s/he have paralysis of both legs? | months | days |
| 40. Was s/he unable to pass urine? | months | days |
| 41. Did s/he pass blood in urine? | months | days |
| 42. Did a dog bite him/her? | months | days |
| 43. Was he/she bitten by another animal or insect? | months | days |
| 44F. If yes, what type of animal/ insect? (Write the name) |  |  |
| 45. Was s/he injured in a road accident? | months | days |
| 46. Did s/he suffer any other accidental injuries before death? | months | days |
| 47. Was s/he injured intentionally by someone? | months | days |

| **Health services used by the deceased during his/her illness in the period leading to death** | | |
| --- | --- | --- |
|  | | Did s/he get medicine here? |
| Was given traditional medicine | | 1-yes  2-no 9-don’t know |
|  | ? order |  |
| Was given modern medicine | | 1-yes  2-no 9-don’t know |
|  | ? order |  |
| Medicine from family | | 1-yes  2-no 9-don’t know |
|  | ? order |  |
| Went to traditional healer | | 1-yes  2-no 9-don’t know |
|  | ? order |  |
| Village health worker | | 1-yes  2-no 9-don’t know |
|  | ? order |  |
| Government dispensary | | 1-yes  2-no 9-don’t know |
|  | ? order |  |
| Government health centre | | 1-yes  2-no 9-don’t know |
|  | ? order |  |

| **Health services used by the deceased during his/her illness in the period leading to death**  **(contd…)** | | |
| --- | --- | --- |
| Government hospital | | 1-yes  2-no 9-don’t know |
|  | ? order |  |
| Medicine from any government health facility | | 1-yes  2-no  9-don’t know |
|  | ? order |  |
| Private dispensary | | 1-yes  2-no 9-don’t know |
|  | ? order |  |
| Private health centre | | 1-yes  2-no 9-don’t know |
|  | ? order |  |
| Private hospital | | 1-yes  2-no 9-don’t know |
|  | ? order |  |
| Private pharmacy | | 1-yes  2-no 9-don’t know |
|  | ? order |  |
| Didn’t get any service | | 1-no service |

| **Other Evidence and Summary of Details** | | |
| --- | --- | --- |
| Death Certificate | | |
| Cause of death | | |
| Burial Permit | | |
| Cause of death | | |
| Post mortem results | | |
| Cause of death | | |
| MCH/ANC Card | | |
| Hospital prescription forms | | |
| Treatment cards | | |
| Hospital discharge forms | | |
| Diagnosis | | |
| Other hospital documents | | |
| Laboratory/cytology results | | |
|  | No evidence | |
| Did a health care worker tell you the cause of death? | | 1-yes  2-no 9-don’t know |
| *If yes, what did s/he say?* | | |
