# Supplementary material for: Core Verbal Autopsy Procedures with Comparative Validation Results from Two Countries
Source: PLoS Med. 2006 Jul 18;3(8):e268. doi: 10.1371/journal.pmed.0030268 (PMC1502154; doi:10.1371/journal.pmed.0030268)
Supplement: Supplementary file 5 [file pmed.0030268.sg003.doc]

| **Field site** | | | |
| --- | --- | --- | --- |
| **Household number** | | | |
| **Date of interview** | Day | Month | Year |
| **Interviewer** | | | |
| **Location identifier 1** | | | |
| **Location identifier 2** | | | |
| **Name of reference person** | | | |
| **Was an appropriate respondent found?**  1- Yes 2-No | | | |
| **Relationship of the respondent to the deceased** | 1-Father 2-Mother 3-Spouse  4-Sibling 5-Child  6-Other relative 7-No relation | | |
| **Did you live with the deceased in the period leading to death?** | | 1-Yes  2-No |  |
| **Name of deceased** | | | |
| **Sex**  1-Male 2-Female | **Date of birth** | | |
|  | Day | Month | Year |
| **Marital Status**  1-never married 2-1 wife/husband 3->1 wife/husband  4-separated 5-divorced 6-widow/er | | | |
| **Education**  0 - None  1 - Some primary  2 - Completed primary  3 - Some secondary  4 - Completed secondary  5 - Post-secondary  6 - Vocational  9 - Don’t know | | | |
| **Place of death** | 1-Home  2-Health facility____________  3-Other | | |
| **Date of Death** | Day | Month | Year |
| **Residential Status**  **The deceased was…**  1 - Resident in the SAVVY area  2 - Body brought home for burial  3 - Home-coming sick | | | |
| **What do you think was the cause of death?**  **(Write exactly as the respondent tells you.)** | | | |
|  | | | |
|  | | | |
|  | | | |
|  | | | |
|  | | | |

| **Previously diagnosed medical conditions:** | | | |
| --- | --- | --- | --- |
|  | Months |  | Months |
| Hypertension |  | Diabetes |  |
| Heart disease |  | TB |  |
| HIV/AIDS |  | Asthma |  |
| Cancer of _______ |  | Leprosy |  |
| Other chronic illness:__________ |  |  | |
| **History of events leading to death** | | | |
|  | | | |
|  | | | |
|  | | | |
|  | | | |
|  | | | |
|  | | | |
|  | | | |
|  | | | |
|  | | | |

| **"I would like to ask you some questions concerning symptoms that the deceased had/showed when s/he was ill. Some of these questions may not appear to be directly related to his/her death. Please bear with me and answer all the questions. They will help us to get a clear picture of all possible symptoms that the deceased had."** | | |
| --- | --- | --- |
| **SYMPTOMS** | | |
| 1. Did s/he have fever? | months | days |
| 2F. If yes, was the fever… | 1-continuous  2-on and off  9-don’t know | |
| 3. Was s/he breathless on light work? | months | days |
| 4. Was s/he breathless when lying flat/ interfering with sleep? | months | days |
| 5. Did s/he have palpitations? | months | days |
| 6. Did s/he have wheezing? | months | days |
| 7. Did s/he have a cough? | months | days |
| 8F. If yes, was the cough… | 1-dry  2-productive  3-with blood  9-don’t know | |
| 9. Did s/he have chest pain? | months | days |
| 10F. If yes, was the pain localised near the sternum? | 1-yes  2-no 9-don’t know | |
| 11F. Did the pain start… | 1-suddenly  2-gradually  9-don’t know | |
| **SYMPTOMS (contd…)** | | |
| 12. Did s/he have diarrhoea? | months | days |
| 13F. If yes, how many times a day? | number | |
| 14. Did s/he have bloody stools? | 1-yes  2-no 9-don’t know | |
| 15. Did s/he have poor appetite? | months | days |
| 16. Did s/he complain of pain on swallowing? | months | days |
| 17. Did s/he have difficulty in swallowing? | months | days |
| 18. Did s/he have headache? | months | days |
| 19. Did s/he pass blood in urine? | months | days |
| 20. Did s/he have pain while passing urine? | months | days |
| 21. Was s/he unable to pass urine? | months | days |
| 22. Did s/he pass urine too many times in a day? | months | days |
| 23. Did s/he have a sensation of pins and needles in the feet? | months | days |
| 24. Did s/he have abdominal pain? | months | days |
| 25F. If yes, was the pain… | 1-upper  2-lower  3-all over abdomen  9-don’t know | |
| 26. Did s/he have abdominal distension? | months | days |
| 27F. If yes, did the distension start… | 1-suddenly within a few days  2-gradually over the weeks  9-don’t know | |
| 28. Did s/he vomit? | months | days |
| 29F. If yes, did s/he vomit blood? | months | days |
| 30. Did s/he have a mass in the abdomen? | months | days |
| 31. Did s/he become mentally confused? | months | days |
| 32. Did s/he have loss of consciousness? | months | days |
| 33F. If yes, did s/he become unconscious… | 1-suddenly  2-gradually as days went by  9-don’t know | |
| 34. Was s/he paralysed on one side of the body? | months | days |
| 35. Did s/he have paralysis of both legs? | months | days |
| 36. Did s/he develop stiffness of the whole body (before death)? | months | days |
| 37. Did s/he have neck pain? | months | days |
| 38. Did s/he have fits? | months | days |
| 39F. If yes, when it was severe, how many times did s/he have fits in a day? | | number |

| **…ASK FOR ALL WOMEN**  **AGED 12 YEARS AND ABOVE…** | | |
| --- | --- | --- |
| 40. How many live births did she have in her entire life? | live births | |
| 41F. If she had one or more live births, how many of those live births died before the age of 5? | deaths | |
| 42. Did she have an ulcer or swelling in the breast? | months | days |
| 43. Did she have excessive vaginal bleeding during her menstrual period? | 1-yes  2-no  9-don’t know | |
| 44. Did she have vaginal bleeding when she was not in her menstrual period? | 1-yes  2-no  9-don’t know | |
| 45F. If yes, did the bleeding persist until death? | 1-yes  2-no 9-don’t know | |
| 46. Did she have abnormal vaginal discharge? | months | days |

| **ASK FOR WOMEN**  **AGED 12 TO 49 YEARS OF AGE…** | |
| --- | --- |
| 47. Was she pregnant? | 1-yes  2-no 9-don’t know |
| 48F. If yes, how many months? | months |
| 49. Did she give birth recently? | 1-yes  2-no 9-don’t know |
| 50F. If yes, how many days before death? | days |
| 51F. Did she have excessive bleeding before delivering the baby? | 1-yes  2-no  9-don’t know |
| 52F. Did she have difficulty in delivering the baby? | 1-yes  2-no 9-don’t know |
| 53F. Did she have difficulty in delivering the placenta? | 1-yes  2-no 9-don’t know |
| 54F. Did she have a prolonged labour? | 1-yes  2-no 9-don’t know |
| 55F. Did she have a caesarian operation for delivery? | 1-yes  2-no 9-don’t know |
| 56F. Did she have a forceps or vacuum delivery? | 1-yes  2-no 9-don’t know |
| 57F. Did she have too much bleeding after delivering the baby? | 1-yes  2-no  9-don’t know |
| 58F. How is the baby? | 1-alive  2-born dead  3-died within 7 days of birth  4- died more than 7 days after birth  5-twins, one died |
| 59. Did she have an induced abortion recently? | 1-yes  2-no 9-don’t know |
| 60F. If yes, how many days before death? | days |

| **…OTHER SIGNS (ALL ADULTS)…** | | |
| --- | --- | --- |
| 61. Did s/he have ankle swelling? | months | days |
| 62. Did s/he have swelling of joints? | months | days |
| 63. Did s/he have weight loss? | months | days |
| 64. Did s/he have mouth sores? | months | days |
| 65. Did s/he look pale? | months | days |
| **…OTHER SIGNS (ALL ADULTS)…**  **(contd…)** | | |
| 66. Did his/her lips grow darker in color? | months | days |
| 67. Did s/he have any skin disease? | months | days |
| 68. Did s/he have puffiness of the face? | months | days |
| 69. Did the eye colour change to yellow (jaundice)? | months | days |
| 70. Was s/he injured in a road accident? | months | days |
| 71. Did s/he suffer any other accidental injuries recently before death? | months | days |
| 72. Was s/he injured intentionally by someone? | months | days |
| 73. Did a dog bite him/her? | months | days |
| 74. Did any other animal or insect bite him/her? (How long before his/her death? | months | days |
| 75F. If yes, what type of animal or insect?  (write the name) | | |
| 76. Was s/he accidentally poisoned (including alcohol)? | months | days |
| 77. Did s/he commit suicide? | 1-yes  2-no 9-don’t know | |

| **Health services used by the deceased during his/her illness in the period leading to death** | | |
| --- | --- | --- |
|  | | Did s/he get medicine here? |
| Was given traditional medicine | | 1-yes  2-no 9-don’t know |
|  | ? order |  |
| Was given modern medicine | | 1-yes  2-no 9-don’t know |
|  | ? order |  |
| Medicine from family | | 1-yes  2-no 9-don’t know |
|  | ? order |  |
| Went to traditional healer | | 1-yes  2-no 9-don’t know |
|  | ? order |  |
| Village health worker | | 1-yes  2-no 9-don’t know |
|  | ? order |  |
| Government dispensary | | 1-yes  2-no 9-don’t know |
|  | ? order |  |
| Government health centre | | 1-yes  2-no 9-don’t know |
|  | ? order |  |
| Government hospital | | 1-yes  2-no 9-don’t know |
|  | ? order |  |
| Medicine from any government health facility | | 1-yes  2-no  9-don’t know |
|  | ? order |  |
| Private dispensary | | 1-yes  2-no 9-don’t know |
|  | ? order |  |
| Private health centre | | 1-yes  2-no 9-don’t know |
|  | ? order |  |
| Private hospital | | 1-yes  2-no 9-don’t know |
|  | ? order |  |
| Private pharmacy | | 1-yes  2-no 9-don’t know |
|  | ? order |  |
| Didn’t get any service | | 1-no service |

| Had the deceased smoked more than 100 cigarettes or 100 grams of tobacco in his/her whole life? | 1-yes  2-no  9-don’t know | |
| --- | --- | --- |
| Did the deceased smoke during one month before death? | 1-yes  2-no 9-don’t know | |
| If no, how long had the deceased stopped smoking before death? | 1-less than 1 month  2-one to six months  3-six months to one year  4-one to five years  5-more than five years  9-don’t know | |
| When the deceased smoked, how many cigarettes or how much tobacco did s/he smoke per day on average? | | number |

| Did the deceased ever drink alcohol? | 1-yes  2-no 9-don’t know |
| --- | --- |
| Did the deceased drink alcohol within the month before death? | 1-yes  2-no 9-don’t know |
| How much did the deceased usually drink? | 1-every day  2-nearly every day  3-four to five times per week  4-once a week  5-two to three times per month  6-once a month  7-four to five times per year  9-don’t know |

| **Other evidence and summary of details** | | |
| --- | --- | --- |
| Death Certificate | | |
| Cause of death | | |
| Burial Permit | | |
| Cause of death | | |
| Post mortem results | | |
| Cause of death | | |
| MCH/ANC Card | | |
| Hospital prescription forms | | |
| Treatment cards | | |
| Hospital discharge forms | | |
| Diagnosis | | |
| Other hospital documents | | |
| Laboratory/cytology results | | |
|  | No evidence | |
| Did a health care worker tell you the cause of death? | | 1-yes  2-no 9-don’t know |
| *If yes, what did s/he say?* | | |
